# Rapidly Progressive IgA Nephropathy in a Patient with Systemic Lupus Erythematosus and Chronic Hepatitis B: A Case Report

**DOI:** 10.3390/reports8040220

**Published:** 2025-10-31

**Authors:** Patrícia Kleinová, Karol Graňák, Tímea Blichová, Matej Vnučák, Ivana Dedinská

**Affiliations:** 1 Transplant-Nephrology Department, University Hospital Martin, Kollárova 2, 036 01 Martin, Slovakia; kleinova.pata@gmail.com (P.K.); timea.blichova@gmail.com (T.B.); vnucak.matej@gmail.com (M.V.); ivana.dedinska@uniba.sk (I.D.); 2 Department of Internal Medicine I, Jessenius Medical Faculty, Comenius University, 036 01 Martin, Slovakia

**Keywords:** rapidly progressive glomerulonephritis, secondary IgA nephropathy, active hepatitis B

## Abstract

**Background and Clinical Significance:** Immunoglobulin A nephropathy (IgAN) is the most common primary glomerulonephritis in adults, typically following a chronic course that often leads to end-stage kidney disease. Rapidly progressive glomerulonephritis is a rare and severe variant of IgAN with a poor prognosis. **Case Presentation:** We present the clinical case of a 68-year-old Caucasian female with a history of systemic lupus erythematosus and untreated chronic hepatitis B, who was admitted to the Transplant-Nephrology Department, University Hospital Martin, with acute kidney injury and nephrotic syndrome accompanied by hematuria. The clinical picture was marked by lower limb oedema and poorly controlled hypertension, both of which responded well to conservative management. Extrarenal causes were excluded through otolaryngologic, stomatologic, and gynecologic assessments, and autoantibody screening was negative. Renal biopsy revealed crescentic glomerulonephritis with endocapillary and mesangial proliferation and IgA deposits. Due to active hepatitis B, initial treatment was limited to corticosteroids. Following a decrease in viral load, pulse therapy with cyclophosphamide was administered, followed by mycophenolic acid; however, renal function did not recover. **Conclusions:** The rapidly progressive form of IgA nephropathy in the context of active hepatitis B presents a rare and challenging clinical case. Management requires a highly individualised, multidisciplinary approach due to the risk of infectious complications and the need to preserve renal function.

## 1. Introduction and Clinical Significance

IgA nephropathy (IgAN) is the most common primary glomerulonephritis in the adult population worldwide, with a notable prevalence in Asian countries. It is a chronic glomerulonephritis in which approximately 20–30% of patients progress to end-stage kidney disease (ESKD) within 10–20 years.

IgAN is associated with other extrarenal diseases, such as gastrointestinal diseases, liver diseases, systemic infectious diseases, autoimmune disorders, and neoplasias—secondary IgAN. Although there are no specific histological signs that differentiate primary from secondary IgAN, the increased frequency of IgAN in association with certain diseases suggests a causal relationship. The most common cause of secondary IgAN is liver disease, with secondary IgAN associated dominantly with hepatitis B among infectious etiologies. The endemic area is again the southeast part of Asia, where IgAN was detected by renal biopsy in approximately 30% of patients with active hepatitis B.

Typically, IgAN presents as asymptomatic with mild to moderate macroscopic hematuria. Rapidly progressive glomerulonephritis (RPGN) with the formation of crescents is a rare and severe clinical manifestation of IgAN with an unfavourable prognosis [1,2,3].

This case is unique due to its complex development from a clinical nephrology perspective, in the context of immunosuppressive treatment for a systemic disease with kidney involvement and reactivation of a chronic infectious disease, requiring antiviral treatment.

## 2. Case Presentation

We present the clinical case of a 68-year-old Caucasian woman referred to our Transplant-Nephrology department at University Hospital Martin from a local hospital with severe nephrotic syndrome, hematuria and acute kidney injury. The patient presented with progressive leg swelling, which she had observed for the past six months, and which significantly worsened one month before our hospitalisation, when she overcame severe SARS-CoV-2 infection—the patient received three doses of the COVID-19 vaccine. Additionally, symptoms included uncontrolled arterial hypertension, generalised fatigue, and mild dyspepsia without vomiting and diarrhoea.

The patient’s medical history included a diagnosis of systemic lupus erythematosus (SLE) diagnosed at the age of 52 (with a predominance of initial skin manifestations such as a rash on the face and neck and with intermittent arthritis of the interphalangeal metacarpophalangeal joints), chronic, untreated hepatitis B and arterial hypertension. The management of SLE had been maintained with a combination of low-dose oral corticosteroids and azathioprine, provided by an outpatient rheumatologist regularly.

Despite triple antihypertensive drugs (angiotensin II receptor blockers, beta-blocker and calcium-channel blocker), her blood pressure control was suboptimal.

The physical examination showed significant oedema of the lower limbs with pitting. Otherwise, the patient was eupnoeic, with cardiopulmonary compensation, and had no respiratory or urinary issues, as well as no history of elevated body temperature. There was no pain in the joints or skin rash. The abdomen was not palpably painful.

Laboratory examinations were performed on admission, and acute kidney injury with nephrotic syndrome was diagnosed [creatinine: 147 µmol/L, estimated glomerular filtration rate: 31 mL/min/1.73 m^2^, albumin: 24.5 g/L, triacylglycerols: 5.1 mmol/L, urine protein/creatinine ratio: 1738.7 g/mol]. Significant microscopic hematuria was detected in urine analysis [229/µL]. eGFR was calculated according to the Chronic Kidney Disease Epidemiology Collaboration (CKD-EPI) formula. The initial tests also detected mild-grade anaemia, mild elevation of inflammatory parameters, hypovitaminosis D, and hypokalemia. The immunological test revealed normal levels of immunoglobulins, as well as normal levels of the complement proteins C3 and C4. Autoantibody screening showed positivity for anti-double-stranded DNA (anti-dsDNA; 47.10 AU/mL), other autoantibodies, including antineutrophil cytoplasmic antibodies (ANCA; both MPO and PR3) and antibodies against GBM, effectively ruling out other common causes of rapidly progressive glomerulonephritis. The panel of autoantibodies was entirely negative, except for the detection of anti-neutrophil cytoplasmic antibodies, with only anti-dsDNA being positive (47.10 AU/mL). In the context of excluding haematological malignancy as a cause of acute kidney injury, the ratio of kappa/lambda free light chains in serum and paraprotein in serum and urine, as determined by electrophoresis, was examined [Table 1].

**Figure 1:**
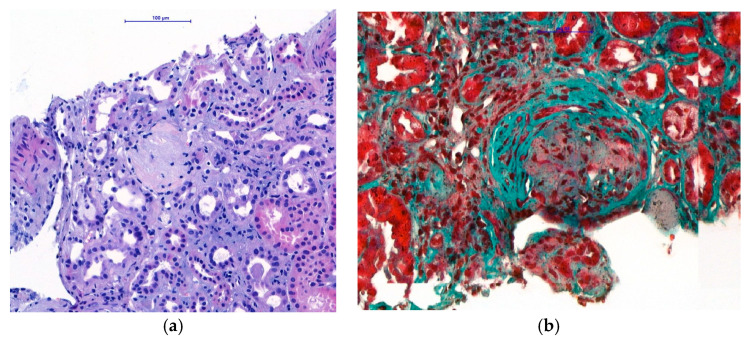
Histological examination of renal biopsy. (**a**) Light microscopy with H&E staining (magnification ×400): globally sclerotic glomeruli. (**b**) Light microscopy with Trichrome staining (magnification ×400): fibrocellular crescents in preserved glomeruli.

Within the differential diagnosis of nephrotic syndrome with hematuria, the ultrasound-guided left kidney biopsy under local anaesthesia was performed the day after admission. The procedure was uncomplicated. The renal histological examination showed 12 glomeruli examined by light microscopy, four of which were globally sclerotic [Figure 1]. The other three glomeruli had a more pronounced sclerotic matrix, with increased endocapillary and mesangial cellularity, as well as thickened basement membranes. The other five glomeruli contained cellular and fibrocellular crescents [Figure 1]; tubulointerstitial injury dominated, with interstitial fibrosis/atrophy ranging from 20% to 30%. Moderate arteriolosclerosis was present in the small arteries without signs of active vasculitis.

**Figure 2:**
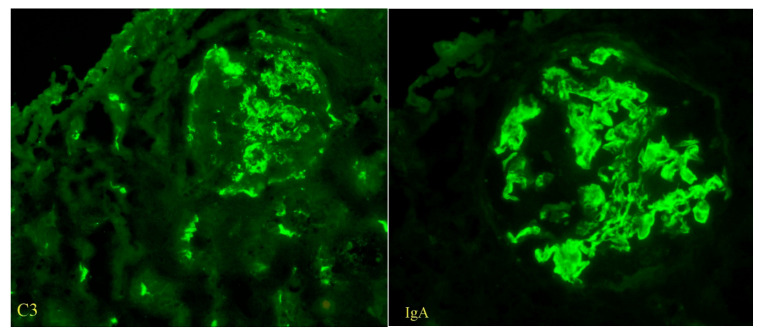
The findings of C3 and IgA deposits, immunofluorescent staining (magnification ×400).

**Figure 3:**
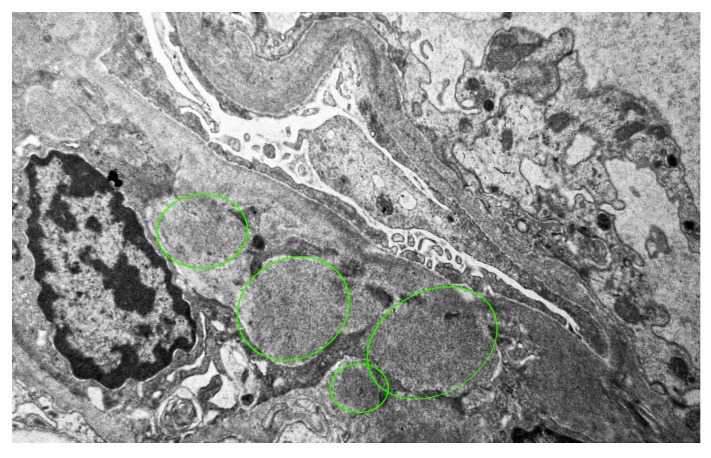
The finding of massive mesangial and subendothelial deposits, electron microscope, magnification ×7000.

Immunofluorescence microscopy revealed dominant mesangial and subendothelial IgA and C3 deposits, with minimal staining for IgM, C1q, fibrinogen, and light chains consistent with IgA nephropathy [Figure 2]. Four glomeruli were examined in electron microscopy, one of which was globally sclerotic. The others showed the presence of massive para-mesangial and subendothelial deposits with a moderate fusion of pedicels of podocytes [Figure 3]. Given the findings of mesangial deposits with increased mesangial cellularity and a predominance of IgA and C3 deposits in immunofluorescence microscopy, this was classified as IgA nephropathy, based on the Oxford classification, with a score of M0 E1 S1 T1 C2. The finding of endo-capillary cellularity and subendothelial deposits, which were dominant, was indicative of lupus nephritis. The presence of crescents in light microscopy was indicative of an aggressive form of IgA nephropathy and, at the same time, an active form of class III lupus nephritis.

Based on the histopathological result of the renal biopsy, rapid deterioration of renal function (creatinemia elevation ≥50% in <3 months) and the presence of humoral SLE activity (anti-dsDNA positivity), we diagnosed rapidly progressive IgA nephropathy in a patient with systemic lupus erythematosus with active lupus nephritis.

Treatment was initiated based on the Kidney Disease: Improving Global Outcomes (KDIGO) guidelines for lupus nephritis class III, consisting of pulse therapy with cyclophosphamide (500 mg every month for 6 months) combined with glucocorticoids. KDIGO recommends similar treatment management for rapidly progressive IgA, but with a higher dose and more frequent administration of cyclophosphamide. Given the history of chronic hepatitis and the risk of infectious complications, we chose to administer cyclophosphamide based on the lupus protocol [4,5].

The patient received pulse therapy with intravenous corticosteroids within 3 days, in a cumulative dose of 1250 mg (500 mg, 500 mg and 250 mg), at the local hospital. Additionally, oral corticosteroids (40 mg) were administered with a gradual dose reduction.

**Figure 4:**
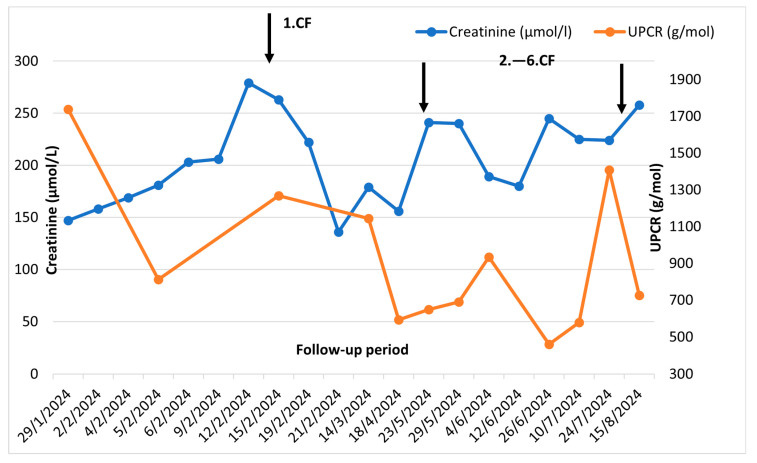
Clinical course before and after cyclophosphamide pulses: UPCR—urine protein/creatine ratio CF—cyclophosphamide.

This therapy led to an improvement in the clinical condition (reduction of swelling of the lower limbs, reduction of weakness) and a decrease in nitrogenous substances, without further progression [Figure 4].

For anaemia of a moderate degree with a positive focal occult bleeding test and subjectively reported dyspeptic complaints, the patient underwent gastrofibroscopy with the finding of interstitial metaplasia of the gastric antrum. High-resolution computer tomography of the lungs was performed to assess the extent of involvement by the underlying disease, without signs of interstitial involvement indicative of SLE. In the differential diagnostic algorithm for extrarenal causes of nephrotic syndrome, otolaryngological, stomatological, and gynaecological examinations were completed with negative findings. Conservative treatment of oedema of lower limbs was started with intravenous diuretics in combination with albumin, with a gradual increase in the dose of diuretics and transition to per os form. The antihypertensive dose was titrated upward.

A high concentration of hepatitis B surface antigen and viremia of hepatitis B virus (900,000 copies/mL) were confirmed by PCR testing five days after immunosuppressive treatment with cyclophosphamide was initiated. Based on serological examinations, a diagnosis of active chronic HBV was established, and antiviral therapy with entecavir was initiated after consultation with an infectious disease specialist the next day. Based on KDIGO recommendations, the dose of entecavir was adjusted in the context of reduced eGFR to 0.5 mg tablet every 72 h [6]. The development of serological and hepatic tests is recorded in Table 2. Due to active chronic HBV infection, it was not possible to continue treatment with cyclophosphamide, rituximab or mycophenolate mofetil. After 2 months of antiviral therapy, there was a significant decrease in HBV viremia (1300 cops/mL). Due to persistent activity of lupus nephritis with worsening of cretinemia and proteinuria, we decided to continue pulse treatment with cyclophosphamide according to the Euro-Lupus protocol (500 mg intravenously every two weeks, 6 times in total). It showed a partial effect, with the most notable improvement in creatinine levels. The maintenance immunosuppressive treatment administered was mycophenolate mofetil, which the patient did not tolerate (gastrointestinal complaints). Therefore, it was changed to the preparation of mycophenolic acid.

The patient was subsequently admitted to the local hospital 3 weeks after the last check-up in our outpatient clinic for deep venous thrombosis of the left lower limb and erysipelas of the right lower limb, and high inflammatory activity (C-reactive protein: 308 mg/L and leucocytosis). We recommended interruption of the immunosuppressive treatment; however, the patient died despite intensive antibiotics and supportive therapy.

Unfortunately, the cause of death was unknown because the patient died in a local hospital. However, even at our request, an autopsy was not performed. Reactivation of HBV infection seems unlikely, since the patient was taking continuous antiviral medication and also during our last outpatient check-up, the viremia was 13 copies/mL even after completing the complete pulse therapy.

## 3. Discussion

Systemic lupus erythematosus is a chronic autoimmune disease, most often manifesting in visceral organs as lupus nephritis. Approximately 10–30% of patients with lupus nephritis will progress to ESKD within 10 years. Based on the histological findings of the renal biopsy, we distinguish five classes of lupus nephritis [7]. Coexistence of lupus nephritis with another autoimmune disease is rare. Although IgAN is the most common primary glomerulopathy in the adult population, its association with lupus nephritis is limited to only a few cases in the available literature [8].

IgA nephropathy is characterised by its gradual progression to ESKD. The etiologies of secondary IgAN are various, including gastrointestinal, respiratory, oncological, and infectious diseases, while liver diseases, especially liver cirrhosis, are considered the most common triggers [9]. Renal involvement in primary IgAN is less severe than in secondary IgAN, as confirmed by Wang et al. [9], who reported that patients with HBsAg-IgAN had a higher rate of IgM and IgG deposition in renal biopsies, accompanied by higher proteinuria and creatinine levels. In most cases, IgAN is clinically characterised by a progressive, asymptomatic course; in 3–5% of cases, the clinical course can be rapidly progressive glomerulonephritis (RPGN) [10]. In such cases, renal function rapidly deteriorates, with the pathognomonic histological finding of glomerular crescents in light microscopy [11]. Studies show that in crescentic IgAN, one-year renal survival rates are only 50%, and 5-year survival rates are only 20% in cases [10].

Our case presents a patient with SLE with lupus nephritis III. class with findings of increased endocapillary and mesangial cellularity, segmental sclerotisation, interstitial fibrosis, tubular atrophy, and crescents. From a histological perspective, it involves the coexistence of lupus nephritis with chronic IgA nephropathy. In the context of the anamnesis of chronic HBV and the high viremia diagnosed by us, we evaluated the condition as secondary IgA nephropathy in chronic hepatitis B.

Despite the significant global incidence of living patients with chronic HBV, according to the available literature, kidney involvement is present in 2–3% of cases. The most common glomerulopathy associated with chronic HBV is membranous glomerulonephritis, followed by membranoproliferative glomerulonephritis, with a minimal occurrence of IgAN. Patients with persistent HBV serology are at risk, especially in endemic regions of Southeast Asia, where an increase in secondary IgAN associated with HBV is expected. According to a 2024 study by Gao et al., chronic, untreated hepatitis B is an independent risk factor for the progression of IgAN [12]. A particularly high-risk group of patients in the context of HBV reactivation are those in whom, despite fully utilised supportive treatment of IgA nephropathy, the renal function does not stabilise and, according to current guidelines, the addition of corticosteroids is recommended for them [12,13]. Authors Shah et al. present a case report of a patient with hepatitis B virus complicated by the development of IgA nephropathy. In their case, however, the viremia was low, without the need for antiviral treatment (3 × 10^3^ copies/mL), and it was also a mild course of IgAN, requiring only supportive treatment [14].

In our case, however, within a few days, the patient experienced a significant deterioration of renal function with increasing proteinuria. Due to the presence of cellular and fibrocellular crescents in 40% of the captured glomeruli, we evaluated the condition as a rapidly progressive form of IgAN.

Because this is a rare case, the treatment procedure is not precisely defined in the professional literature. Antiviral therapy should include the use of a nucleoside/nucleotide analogue, and immunosuppression consists of pulsed steroid therapy with or without cyclophosphamide or rituximab [15].

Since rapidly progressive glomerulonephritis GN has an unfavourable prognosis with rapid deterioration of renal functions, despite the non-existent treatment protocol for RPGN in patients with IgA nephropathy, we decided to administer a pulse of intravenous cyclophosphamide (500 mg). Combined treatment (cyclophosphamide and corticoids) improved the patient’s clinical condition (reduction in swelling, return of appetite) and cessation of progression of renal parameters. However, after receiving the HBV PCR results, we discontinued pulse therapy with cyclophosphamide.

We initiated entecavir treatment, accompanied by a gradual reduction in oral corticosteroid dose. Following antiviral treatment, viremia decreased, allowing us to continue cyclophosphamide administration in a cumulative dose of 6 pulses, with partial restoration of renal parameters. A preparation of mycophenolic acid was subsequently added to the chronic treatment, which was replaced by mycophenolate mofetil due to intolerance. A similar case of a patient with RPGN in IgAN was reported by Patel et al., in which, instead of cyclophosphamide, five cycles of plasmapheresis were selected as the next treatment step after corticoid pulses. However, the patient did not recover her renal functions, with the need for hemodialysis [16].

Only one case of RPGN associated with IgAN in chronic HBV has been reported in the literature. In this case, unlike our patient, after histological confirmation of RPGN, therapy was postponed due to the absence of exclusion of an active infectious aetiology. In comparison to our case, this patient was treated with entecavir for an extended period. Wiese J et al., after pulse steroid treatment, the patient was prescribed mycophenolate mofetil at a dose of 1 g twice a day for fear of HBV reactivation during treatment with cyclophosphamide or rituximab. As in the previous case, in this patient, despite immunosuppressive treatment, renal function deteriorated with the need for their replacement [17].

From a medical management perspective, this represents a challenging and rare clinical scenario that every nephrologist may eventually encounter. On the one hand, there is the urgent need to treat rapidly progressive glomerulonephritis to preserve kidney function and avoid renal replacement therapy; on the other hand, the presence of active hepatitis B restricts both the timing and intensity of immunosuppressive treatment.

This case highlights the importance of early identification of chronic viral infections before initiating immunosuppression. Whenever possible, antiviral therapy should be commenced before or concomitantly with immunosuppressive treatment to minimise the risk of HBV reactivation and hepatic complications. Another important message for clinical practice is the need for strict monitoring of HBV viremia levels in these patients, despite antiviral treatment, before administering any intravenous immunosuppression. Close and continuous monitoring of HBV DNA levels, liver enzymes, and renal function throughout the treatment course is essential to guide therapeutic decisions and ensure patient safety.

Management of such cases should follow a multidisciplinary approach involving nephrologists, hepatologists, and infectious disease specialists to balance the benefits of immunosuppression against the risks of viral reactivation. Despite early intervention and appropriate therapeutic adjustments, RPGN remains associated with high morbidity and mortality, and current literature still lacks evidence-based guidelines for these complex situations. Therefore, our case contributes valuable clinical insight into the coordination of antiviral and immunosuppressive strategies in patients presenting with concurrent immune-mediated and infectious pathologies.

## 4. Conclusions

Rapidly progressive IgA nephropathy secondary to hepatitis B represents a rare but clinically significant challenge. This case highlights the importance of early diagnosis of HBV infection in patients who may require immunosuppressive therapy. Initiation of antiviral treatment before immunosuppression is essential to reduce the risk of viral reactivation and improve both hepatic and renal outcomes.

## Figures and Tables

**Table 1:** Admission laboratory parameters: TAGs—triacylglycerols, LDL-cholesterol—low-density lipoprotein cholesterol, HDL-cholesterol—high-density lipoprotein cholesterol, Ig—immunoglobulin, WBCs—white blood cells, UPCR—urine protein/creatine ratio, eGFR—estimated glomerular filtration rate, HBV—hepatitis B virus, PCR—polymerase chain reaction; C—reactive protein.

Laboratory Parameter	Values
Creatinine (µmol/L)	**147** [59…104]
Urea (mmol/L)	**16** [2.8…7.2]
Uric acid (µmol/L)	**475** [208…428]
Total protein (g/L)	45.5 [66.0….83.0]
Albumine (g/L)	**24.5** [35.0…52.0]
Total cholesterol (mmol/L)	4.95 [<5.17]
TAGs (mmol/L)	**5.01** [0.40…1.70]
LDL-cholesterol (mmol/L)	2.03 [1.00…3.30]
HDL-cholesterol (mmol/L)	1.16 [1.03…2.00]
Kappa free light chain (mg/L)	**30.54** [3.30…19.40]
Lambda free light chain (mg/L)	**17.06** [5.71…26.30]
Kappa/Lambda ratio	**1.79** [0.26…1.65]
IgG (g/L)	5.070 [5.400…18.220]
IgA (g/L)	1.142 [1.010…6.450]
IgM (g/L)	0.740 [0.220…2.400]
Complement C3 (g/L)	0.94 [0.9…1.80]
Complement C4 (g/L)	0.16 [0.10…0.40]
anti-dsDNA (AU/mL)	**47.10** [<…20]
Haemoglobin (g/L)	**110** [120…155]
Platelets (10^9^/L)	181 [140…400]
WBCs (10^9^/L)	**12.9** [3.90…10.00]
UPCR (g/mol)	**1738.7** [<15]
eGFR (mL/min/1.73 m^2^)	**31** [64–104]
Hematuria (erythrocytes/µL)	**229** [<15]
HBV PCR (IU/mL)	**902,000**
CRP (mg/L)	1 [<5]

**Table 2:** Development of serology and liver enzymes: HBsAg—hepatitis B surface Antigen, anti-HBs—antibody to hepatitis B surface antigen, anti-HBc IgG—IgG antibody to hepatitis B core antigen, anti-HBc IgM—IgM antibody to hepatitis B core antigen, DNA—deoxyribonucleic acid, GGT—gamma-glutamyl transferase, AST—aspartate transaminase, ALT—alanine transaminase; ALP—alkaline phosphatase. * antiviral treatment with entecavir 0.5 mg tablet every 72 h.

	01/2024 Before Treatment *	02/2024 1st Month of Treatment *	04/2024 3rd Month of Treatment *
HbsAg (IU/L)	**2016.20** [<1.00]	**1902.64** [<1.00]	**1850.40** [<1.00]
anti-HBs (IU/L)	3.70 [<10.00]	2.70 [<10.00]	3.80 [<10.00]
anti-HBc IgG (IU/L)	**211.53** [<0.90]	**187.22** [<0.90]	**166.81** [<0.90]
anti-HBc IgM (IU/L)	0.13 [<0.80]	0.33 [<0.80]	0.15 [<0.80]
HBV DNA (cop/mL)	**902,000**	**20,300**	**1300**
GGT (U/L)	**141.6** [4.2…37.8]	**120.6** [4.2…37.8]	**40.2** [4.2…37.8]
AST (U/L)	21 [6…36]	19.2 [6…36]	22.8 [6…36]
ALT (U/L)	17.4 [6…36]	16.2 [6…36]	29.4 [6…36]
ALP (U/L)	45 [30…129]	34.8 [30…129]	28.2 [30…129]

## Data Availability

The original contributions presented in this study are included in this article; further inquiries can be directed to the corresponding author.

## Abbreviations

ALP	alkaline phosphatase
ALT	alanine transaminase
ANCA	antineutrophil cytoplasmic antibodies
anti-HBc IgG	IgG antibody to hepatitis B core antigen
anti-HBc IgM	IgM antibody to hepatitis B core antigen
anti-dsDNA	anti-double-stranded DNA
AST	aspartate transaminase
CF	cyclophosphamide
CKD-EPI	Chronic Kidney Disease Epidemiology Collaboration
CRP	C-reactive protein
DNA	deoxyribonucleic acid
eGFR	estimated glomerular filtration rate
ESKD	end-stage kidney disease
GGT	gamma-glutamyl transferase
HBsAg	hepatitis B surface Antigen
HBV	hepatitis B virus
HDL-cholesterol	high-density lipoprotein cholesterol
Ig	immunoglobulin
IgAN	Immunoglobulin A nephropathy
KDIGO	Kidney Disease: Improving Global Outcome
LDL-cholesterol	low-density lipoprotein cholesterol
PCR	polymerase chain reaction
RPGN	rapidly progressive glomerulonephritis
SLE	systemic lupus erythematosus
TAGs	triacylglycerols
UPCR	urine protein/creatine ratio
WBCs	white blood cells
